# Pediatric Patient and Parental Anxiety and Impressions Related to Initial Gastrointestinal Endoscopy: A Japanese Multicenter Questionnaire Study

**DOI:** 10.1155/2015/797564

**Published:** 2015-08-31

**Authors:** Shin-ichiro Hagiwara, Yoshiko Nakayama, Manabu Tagawa, Katsuhiro Arai, Takashi Ishige, Takatsugu Murakoshi, Hiroko Sekine, Daiki Abukawa, Hiroyuki Yamada, Mikihiro Inoue, Takeshi Saito, Takahiro Kudo, Yoshitaka Seki

**Affiliations:** ^1^Division of General Pediatrics, Saitama Children's Medical Center, 2100 Magome, Iwatsuki-ku, Saitama 339-8551, Japan; ^2^Department of Pediatrics, Shinshu University School of Medicine, 3-1-1 Asahi, Matsumoto, Nagano 390-8621, Japan; ^3^Department of Pediatrics, University of Tsukuba Hospital, 2-1-1 Amakubo, Tsukuba, Ibaraki 305-8576, Japan; ^4^Division of Gastroenterology, National Center for Child Health and Development, 2-10-1 Okura, Setagaya, Tokyo 157-8535, Japan; ^5^Department of Pediatrics, Gunma University Graduate School of Medicine, 3-39-22 Showa-machi, Maebashi, Gunma 371-0034, Japan; ^6^Department of Gastroenterology, Tokyo Metropolitan Children's Medical Center, 2-8-29 Musashidai, Fuchu City, Tokyo 183-8561, Japan; ^7^Children's Center for Health and Development, Saiseikai Yokohama Tobu Hospital, 3-6-1 Shimosueyoshi, Tsurumi-ku, Yokohama City, Kanagawa 230-0012, Japan; ^8^Department of General Pediatrics, Miyagi Children's Hospital, 4-3-17 Ochiai, Aoba-ku, Sendai, Miyagi 989-3126, Japan; ^9^Department of Pediatric Gastroenterology, Nutrition and Endocrinology, Osaka Medical Center and Research Institute for Maternal and Child Health, Murodo-cho 840, Izumi-C, Osaka 594-1101, Japan; ^10^Department of Gastrointestinal and Pediatric Surgery, Mie University Graduate School of Medicine, 2-174 Edobashi, Tsu, Mie 514-8507, Japan; ^11^Department of Pediatric Surgery, Graduate School of Medicine, Chiba University, 1-8-1 Inohana, Chuo-ku, Chiba City, Chiba 260-8677, Japan; ^12^Department of Pediatrics, Juntendo University Faculty of Medicine, 2-1-1 Hongo, Bunkyo-ku, Tokyo 113-8421, Japan; ^13^The Department of Pediatrics and Child Health, Kurume University School of Medicine, 67 Asahimachi, Kurume City, Fukuoka 830-0011, Japan

## Abstract

*Objective. *To assess anxiety among pediatric patients and their parents related to initial gastrointestinal endoscopy. *Methods.* Patients aged <19 years undergoing initial gastrointestinal (GI) endoscopy and their parents were invited to complete a self-administered questionnaire related to endoscopy in 13 institutions in Japan. *Results.* The subjects were 128 children, aged 1 month to 17 years. Forty-eight patients (37.5%) underwent esophagogastroduodenoscopy (EGD), 32 (25%) underwent colonoscopy (CS), 39 (30.5%) underwent both EGD and CS, 3 (2.3%) underwent balloon enteroscopy (BE), 3 (2.3%) underwent capsule endoscopy (CE), and 3 (2.3%) underwent CE and other endoscopic procedures. In the preendoscopy questionnaire, the most common concerns of the patients and parents before undergoing the procedure were “Pain” (45% of the patients underwent EGD or BE via the oral approach, and 52% of the patients underwent CS or BE via the anal approach) and “Procedural accidents related to the endoscopy” (63% of parents). In the postendoscopy questionnaire, the most common difficulty that patients and parents actually experienced before and after undergoing the procedure was “Hunger.” *Conclusion.* A preparatory intervention including an explanation regarding specific concerns before initial GI endoscopy, which this study revealed, could reduce anxiety experienced by both pediatric patients and parents.

## 1. Introduction

Gastrointestinal (GI) endoscopy is a useful modality for the diagnosis and treatment of various pediatric gastrointestinal disorders. As with adults, pediatric patients and their parents often exhibit anxiety about GI endoscopy in daily medical practice because of the requirement of sedation or general anesthesia for pain and discomfort associated with the examination and because of the potential for rare procedural accidents such as bleeding or intestinal perforation [1–6]. Although attending physicians strive to obtain consent for GI endoscopy from pediatric patients and their parents by carefully explaining the purposes, methods, and possible procedural complications of endoscopy and all medical staff involved in GI endoscopy routinely work to relieve the anxiety of both patients and parents, there is no report regarding the anxiety and satisfaction of Japanese pediatric patients and their parents related to initial GI endoscopy. To create guidelines for pediatric GI endoscopy to ensure procedural safety and effectiveness, the Japanese Pediatric Gastrointestinal Endoscopy Guideline Working Group was established in December 2013. This working group planned a multicenter questionnaire study of pediatric GI endoscopy to design a system from the viewpoints of the patients and parents to lower anxiety among children who require GI endoscopy.

## 2. Materials and Methods

This prospective study was conducted between January and August 2014 in 13 member institutions of the Japanese Pediatric Gastrointestinal Endoscopy Guideline Working Group, which comprised seven university colleges, five children hospitals, and one general hospital. This study was conducted in accordance with the Helsinki Declaration and approved by the Ethics Committee of each facility. Pre- and postprocedural interventions were different among each institution.

The inclusion criteria were as follows: (i) patient age < 19 years at the time of GI endoscopy (esophagogastroduodenoscopy (EGD), colonoscopy (CS), balloon enteroscopy (BE), capsule endoscopy (CE), and endoscopic retrograde cholangiopancreatography (ERCP)) and (ii) consent agreement to participate both from the patients (elementary school students or above) and their parents. In case of preschool-aged children, only the parents completed the questionnaire. Patients meeting the following criteria were excluded: severe mental and physical disabilities.

### 2.1. Study Design

This investigator-initiated study was a prospective observational survey that used a self-administered questionnaire with no involvement of the industry in the design, conduct, funding, or analysis of the results. Informed consent was obtained from the patient and parents before data collection with a questionnaire regarding GI endoscopy. After the attending physician explained the procedure, preendoscopy questionnaires were completed just before the patient underwent the procedure. Both the patients and parents completed postendoscopy questionnaires on the day of endoscopy. If the patient showed a delayed recovery from anesthesia, the patient's postendoscopy questionnaire was completed on the following day. The completed questionnaires were sealed in an envelope and passed on to a medical service provider other than the attending physician. The recovered questionnaires were then directly sent to an analyst without opening the envelope.

### 2.2. The Variety of Questionnaires

The pre- and postendoscopy questionnaires for the parents were prepared in common regardless of the endoscopy type. Three types of questionnaires were distributed to the patients according to the type of endoscopy: (A) EGD/BE via the oral approach/ERCP; (B) CS/BE via the anal approach; and (C) CE. If the patient underwent both EGD and CS, two types of questionnaires (A and B) were distributed. In the pre- and postendoscopy questionnaires, standard answers for each question were provided and respondents selected the applicable answers.

### 2.3. Questions before Endoscopy

The preendoscopy questionnaire included the following: “presence or absence of anxiety,” “reasons why patients/parents did not become anxious,” “the persons whom patients/parents will consult about the procedure,” “the concerns patients/parents felt before the procedure,” and “detailed explanations of what patients/parents wish should be provided.”

### 2.4. Questions after Endoscopy

The postendoscopy questionnaire included the following: “what patients/parents found difficult about preparation,” “what patients/parents found difficult after undergoing endoscopy,” and “impression of the procedure, which was rated on a 5-point scale (1 = very easy; 2 = easy; 3 = fair; 4 = difficult; 5 = very difficult).”

### 2.5. Data Analysis

Collected data were coded for statistical analysis. Continuous variables are presented as mean ± standard deviation. Categorical variables are presented as numbers and percentages. The percentages of answers indicating the use of laxatives, antifoaming agents, and pharyngeal anesthesia were calculated from the total number of patients who underwent each pretreatment medication. In this study, we analyzed the collected data of the patients and parents who underwent initial GI endoscopy.

## 3. Results

### 3.1. Patient Demographics

Between January and August 2014, a total of 300 consecutive patients who underwent pediatric GI endoscopy and their parents participated in this study. Of these, 128 patients underwent initial GI endoscopy, whereas 172 patients underwent GI endoscopy twice or more. In this study, the collected data of 128 patients were analyzed. The characteristics of the patients who underwent initial GI endoscopy are shown in Table 1. There were 70 males (55%) and 58 females (45%) aged 1 month to 17 years. Forty-eight patients (37.5%) underwent EGD; 32 (25%) underwent CS; 39 (30.5%) underwent both EGD and CS; 1 (0.7%) underwent BE via the oral approach; 2 (1.5%) underwent BE via the anal approach; 3 (2.3%) underwent CE; 2 (1.5%) underwent CE, EGD, and CS; and 1 (0.7%) underwent CE, EGD, and BE via the anal approach. Forty-seven patients (36.7%) underwent general anesthesia, 78 (60.9%) underwent intravenous anesthesia, and 3 (2.3%) underwent the procedure without sedation. Although 2 patients who underwent EGD (1.5%) experienced vomiting after the procedure, there was no severe procedural accident associated with both endoscopy and anesthesia. There were no reports about incomplete procedures secondary to incomplete sedation.

### 3.2. Preendoscopy Questionnaire Response from Patients

In 128 patients, 77% of the patients who underwent EGD or BE via the oral approach (EGD group) and 75% of the patients who underwent CS or BE via the anal approach (CS group) reported that they were concerned about undergoing endoscopy. Preprocedural concerns are shown in Table 2(a). The most common concerns in the EGD group were as follows: (1) pain during the procedure (45%); (2) will the procedure be completed while I am sleeping? (37%); and (3) how soon after the procedure can I eat? (34%). The most common concerns in the CS group were as follows: (1) pain during the procedure (52%); (2) how soon after the procedure can I eat? (41%); (3) will I feel ill after the procedure? (41%); and (4) is it necessary to take laxatives? (41%). The reasons why the patients were free from uncertainty are shown in Table 2(b). In total, 33% of the patients from the EGD group and 38% from the CS group replied that the clarification provided by the attending physician relieved anxiety.

In 128 patients, 25% of the patients from the EGD group and 26% from the CS group wanted a more detailed explanation of the endoscopic procedure beforehand. The most common detailed explanations that the EGD group wished for were as follows: (1) the size of the endoscope (50%); (2) flavor and the amount of pretreatment medication (43%); (3) how to fall asleep during the procedure (43%); and (4) meals before and after the procedure (43%) (Table 2(c)). The most common detailed explanations that the CS group wished for were as follows: (1) Flavor and the amount of pretreatment medicine (64%); (2) How to perform endoscopy (50%); (3) In what type of rooms will the endoscopy be performed? (42%); (4) Meals before and after the procedure (42%); and (5) How to spend time during the procedure (42%) (Table 2(c)).

Of the 6 patients who underwent CE, 3 answered the questionnaire, of which 2 were concerned about “Pain.”

### 3.3. Preendoscopy Questionnaire Response from Parents

Approximately 60% of parents reported that they were concerned about the endoscopic procedure. Preprocedural concerns are shown in Table 2(a). The most common concerns of parents were as follows: (1) Procedural accidents related to endoscopy (63%) and (2) Procedural accidents related to sedation or anesthesia (56%). Approximately 32% of the parents wanted a more detailed explanation regarding endoscopy before the procedure. The reasons why the parents were free from uncertainty are shown in Table 2(b). The majority (81%) of the parents replied that the explanation by the attending physician relieved their anxiety as well as that of the patients.

The most common detailed explanations were as follows: (1) feedback from the patients and parents who underwent endoscopy previously (42%); (2) the size of the endoscope (32%); (3) how to spend time during the procedure (26%); and (4) procedural accidents related to endoscopy (26%) (Table 2(c)).

### 3.4. Postendoscopy Questionnaire Response from Patients

The difficulties that the patients experienced before undergoing endoscopy are shown in Table 3(a). Patients in the EGD and CS groups provided the following answers as difficulties before endoscopy: (1) Hunger before the procedure (EGD group, 60%; CS group, 63%) and (2) Pain associated with peripheral vascular access or blood testing (EGD group, 27%; CS group, 35%).

Approximately 63% of patients (*N* = 46) who underwent pharyngeal anesthesia replied that they found pharyngeal anesthesia difficult. Of the patients (*N* = 46) who took laxatives, 72% replied with the large amount of laxatives and 70% replied with terrible taste of laxatives as difficulties before endoscopy.

The most common difficulties after undergoing endoscopy are shown in Table 3(b). In the EGD group, the patients experienced the following difficulties: (1) Hunger after the procedure (44%) and (2) Dizziness after the procedure (41%). In the CS group, the patients experienced the following difficulties: (1) Hunger after the procedure (54%) and (2) Thirst (40%) (Table 3(a)). Regarding impressions about the procedure, which was rated on a 5-point scale, approximately 32% of patients in the EGD group and 44% of those in the CS group rated the procedure difficult or very difficult (4 and 5 points, resp.).

Of the 6 patients who underwent CE, 3 answered the questionnaire, of which 2 replied with “Hunger” as difficulties before and after CE.

### 3.5. Postendoscopy Questionnaire Responses from the Parents

The difficulties that the parents experienced before undergoing endoscopy are shown in Table 3(a). The parents answered the following difficulties: (1) Hunger before the procedure (61%) and (2) Pain associated with peripheral vascular access or blood testing (25%). Approximately 28% of parents (*N* = 46) of the patients who underwent pharyngeal anesthesia replied that pharyngeal anesthesia was difficult for their children. Of the parents (*N* = 46) whose children took laxatives, 96% replied with the large amount of laxative and 91% replied with the terrible taste of laxatives as difficulties for the patients. The difficulties that the parents experienced after undergoing endoscopy are shown in Table 3(b). The parents were most concerned with (1) Hunger after the procedure (51%), (2) Throat soreness (48%), and (3) Thirst after the procedure (31%). Regarding impressions about the procedure, which was rated on a 5-point scale, approximately 57% of parents replied that the procedure was difficult or very difficult (4 and 5 points, resp.).

## 4. Discussion

In this study, we made two important clinical observations. First, the most common concern of the patients was “Pain,” and the most common concern of the parents was “Procedural accidents related to the endoscopy” before undergoing the procedure. Second, the difficulty that both patients and parents actually experienced before and after undergoing the procedure was “Hunger.”

Approximately 80% of the patients and 60% of the parents were anxious before GI endoscopy. McEntire et al. reported that 56% of adult patients undergoing elective colonoscopy experienced moderate to severe anxiety [3]. The results of our study revealed that approximately 80% of patients experienced anxiety before undergoing GI endoscopy, which indicates that children were more likely to be anxious than adults. Moreover, this study showed that more than half of the parents were anxious about the procedures. The explanation by the attending physician was the main reason why patients and parents had lower anxiety before the procedure. A previous study of adults showed findings similar to our study [7]; thus, it is important for the attending physician to carefully explain aspects of the examination. Some studies reported that more adult patients who underwent GI endoscopy were most anxious about pain and the result of the procedure [3, 4]. On the other hand, the results of the present study revealed that many pediatric patients were anxious about pain related to endoscopy, similar to adults. Furthermore, the parents were concerned about child-specific anxiety. Pediatric patients tended to be more anxious about the endoscopic procedure itself rather than the underlying disease, in contrast to adults. Therefore, the medical staff should explain the procedures in consideration of these findings. Voiosu et al. reported that 70.2% of adult patients who underwent colonoscopy considered the information provided prior to the procedure to be insufficient [8]. Khour et al. reported that patient information on medication intake before pediatric digestive endoscopy was insufficient and that patients would appreciate a more detailed description on the type of sedation during the procedure [9]. In our study, only 30% of both patients and parents replied that they desired a more detailed description of the procedure, which revealed that the participating institutions offered thorough explanations.

Some studies have investigated methods for the psychological preparation of children undergoing endoscopy. Mahajan et al. reported that the psychological preparation before endoscopy significantly decreased anxiety in both pediatric patients and their parents [10]. Tanaka et al. reported that the mean level of salivary chromogranin, which is a marker of the mental stress response, was significantly lower after colonoscopy than that before the procedure among children who received an explanation using a guidebook and were allowed to play with dolls and medical instruments [11]. Although age- and developmental-appropriate psychological preparation before the procedure is important [12, 13], it is difficult to achieve ideal preparation in all facilities in light of the situation in Japan because the number of medical staffs (such as child life specialist) involved in pediatric endoscopy is small. Some studies reported that information provided by endoscopic brochures and leaflets contributed to the reduction of patient's anxiety [14, 15]. In a pediatric population, Riddhiputra and Ukarapol reported that preparatory intervention using systemic visual illustration of the technical procedures for children undergoing gastrointestinal endoscopy could significantly reduce parental anxiety [16].

Hence, the anxiety before GI endoscopy can be reduced further by offering visual illustrations that contain the answers to their worries revealed in this study.

The difficulty that patients and parents experienced before and after undergoing the procedure was “Hunger,” which was an inevitable consequence for ensuring the safety of the procedures. This result shows that participating institutions conducted appropriate GI endoscopy, including the procedure itself and anesthesia. To the best of our knowledge, this is the first report describing feedback from patients and parents before and after GI endoscopy. Most patients in the EGD and CS groups considered that “Hunger” was the most difficult issue before and after endoscopy. Because fasting for GI endoscopy is essential, it is not possible to avoid the feeling of “Hunger.” The feeling of “Hunger” before procedures may be reduced by ensuring priorities on list for young children, morning procedures, or timely scheduled procedures. Additionally we also considered the feeling of “Hunger” after procedures. Each facility has specific criteria regarding when the patient can begin drinking fluids (such as water) after the recovery of consciousness; thus, a consensus for the start time to drink fluids after procedures should be reached to relieve the feeling of “Hunger” after endoscopy.

With respect to bowel preparation, many patients who took laxatives before procedures answered that they found it difficult to ingest large amounts and complained about the terrible taste. Although several methods to introduce laxatives to pediatric patients before colonoscopy have been proposed, there is no gold standard and preparatory methods differ among institutions [17, 18]. It is not possible to apply the findings of international trails regarding laxative administration before colonoscopy in Japan because the types of laxatives vary among countries. Therefore, further research is warranted to identify methods for pediatric patients to drink more easily after endoscopy by reducing the amount of laxatives and improving the taste of laxatives used in Japan. Many patients and parents replied that they were most concerned about the safety of endoscopy. Therefore, medical staff performing pediatric GI endoscopy should consider the anxiety of patients and parents before endoscopy.

There were some limitations to this study that should be mentioned. First, the preparatory methods related to GI endoscopy differed among facilities. Some facilities use endoscopic instructions for adults as a substitute and some used procedures intended for children. In the facilities that use procedures specifically for children, the anxiety of the patients and parents may be reduced. Second, this study did not measure anxiety levels about procedures. Thus, the degree of anxiety among participants could not be objectively evaluated, and it cannot be compared with other studies with respect to anxiety levels. Third, the subjects of this study were Japanese. Therefore, there might be differences about the items of concern related to pediatric GI endoscopy among countries with different cultures. Further research is desirable.

## 5. Conclusions

The results of this study clearly revealed two important clinical observations. First, the most common concern of the patients was “Pain” and the most common concern of the parents was “Procedural accidents related to the endoscopy” before undergoing the procedure. Second, the difficulty that both patients and parents actually experienced before and after undergoing the procedure was “Hunger.” It is possible that anxiety before endoscopy experienced by both patients and parents is reduced further by providing explanations regarding specific concerns based on the results of the questionnaire revealed in this study. Future studies should be undertaken to further reduce anxiety based on the results of this study.

## Figures and Tables

**Table 1:** Characteristics of the patients.

Characteristics (*n* = 128)	
Age (years)	
Mean (SD)	9.8 (4.5)
Range	1 month–17 years
Gender	
Male, *n* (%)	70 (55)
Female, *n* (%)	58 (45)
Weight (kg)	
Mean (SD)	32.7 (14.9)
Types of endoscopy (1)	
EGD, *n* (%)	48 (37.5)
CS, *n* (%)	32 (25)
EGD + CS, *n* (%)	39 (30.5)
BE via the oral approach, *n* (%)	1 (0.7)
BE via the anal approach, *n* (%)	2 (1.5)
CE, *n* (%)	3 (2.3)
CE + EGD + CS, *n* (%)	2 (1.5)
CE + EGD + BE via the anal approach, *n* (%)	1 (0.7)
Types of endoscopy (2)	
Selective endoscopy, *n* (%)	126 (98.4)
Emergent endoscopy, *n* (%)	2 (1.6)
Preparation	
Use of antifoaming agents, *n* (%)	36 (28)
Use of laxatives, *n* (%)	46 (36)
Sedation	
General anesthesia, *n* (%)	47 (36.7)
Intravenous anesthesia, *n* (%)	78 (60.9)
Without sedation, *n* (%)	3 (2.3)
Complications	
Related to endoscopy, *n* (%)	2 (1.1)
Related to sedation or anesthesia, *n* (%)	0 (0)

EGD, esophagogastroduodenoscopy; CS, colonoscopy; BE, balloon enteroscopy; CE, capsule endoscopy.

**Table:** (a) What are your concerns about endoscopy?

	EGD group (%)	CS group (%)	Parents (%)
Pain during the procedure	45	52	35
How soon after the procedure can I (my child) eat?	34	41	21
To insert an intravenous line	9	9	9
Will the procedure be completed while I am (my child is) sleeping?	37	32	23
Will I feel ill after the procedure?	29	41	—^*∗*^
When can I (my child) go to school?	15	20	4
To undergo pharyngeal anesthesia^†^	26	—	13
It is necessary to take antifoaming agents^†^	5	—	14
It is necessary to take laxatives^†^	—	41	46
Procedural accidents related to endoscopy	—	—	63
Procedural accidents related to sedation or anesthesia	—	—	56
Some disease may be found	20	26	34
Influence of exposure	—	—	5
Years of experience of the operator in endoscopy	—	—	21
There is nothing to be concerned	14	9	7

EGD group (*N* = 64); CS group (*N* = 53); parents (*N* = 117).

Multiple answers are allowed.

^†^Calculated from the total number of patients who underwent each pretreatment medication.

^*∗*^N/A.

**Table:** (b) Reasons why you did not become anxious

	EGD group (%)	CS group (%)	Parents (%)
Explanation by the attending physician	33	38	81
Explanation by the child life specialist	6	7	0
Explanation by family	13	23	2
Be accustomed to medical test other than GI endoscopy	20	23	2
I had undergone endoscopy previously	—^*∗*^	—	27

EGD group (*N* = 15); CS group (*N* = 13); parents (*N* = 48).

Multiple answers are allowed.

^*∗*^N/A.

**Table:** (c) Detailed explanation of what you wish should be provided to you

	EGD group (%)	CS group (%)	Parents (%)
Reasons for the procedure	25	14	0
How to perform endoscopy	31	50	21
How thick is the endoscope?	50	28	32
Flavor and the amount of pretreatment medication	43	64	8
How to fall asleep during the procedure	43	28	21
Family stands by me during the procedure?	25	21	—^*∗*^
In what type of rooms will the endoscopy be performed?	31	42	—
Explanation by using pictures or dolls	18	7	5
Meals before and after the procedure	43	42	13
How to spend time during the procedure	37	42	26
I need someone to talk about anxiety about the procedure	6	7	—
Feedback from the patients and parents who underwent endoscopy previously	31	35	42
Alternative test to GI endoscopy	—	—	18
Procedural accidents related to endoscopy	—	—	26
Procedural accidents related to sedation or anesthesia	—	—	21
Influence of exposure	—	—	10
Psychological care by medical staff except physicians	—	—	13

EGD group (*N* = 16); CS group (*N* = 14); parents (*N* = 38).

Multiple answers are allowed.

^*∗*^N/A.

**Table:** (a) What did you find difficult about preparation?

	EGD group (%)	CS group (%)	Parents (%)
I (my child) could not sleep for worrying about endoscopy	11	15	18
Hunger before the procedure	60	63	61
Pain associated with peripheral vascular access or blood test	27	35	25
Diet before the procedure tasted bad	—^*∗*^	4	3
I found it no trouble (my child did not look so bad)	18	13	13

EGD group (*N* = 62); CS group (*N* = 48); parents (*N* = 119).

^*∗*^N/A.

**Table:** (b) What did you find difficult after undergoing endoscopy?

	EGD group (%)	CS group (%)	Parents (%)
Awakening during the procedure	2	4	4
Feel terrible during the procedure	6	3	—^*∗*^
Abdominal pain during the procedure	9	10	—
Throat soreness	39	—	48
Nasal soreness	2	—	—
Flatulence after the procedure	6	10	5
Abdominal pain after the procedure	20	19	12
Dizziness after the procedure	41	29	18
Long time to recover from anesthesia	6	8	13
Hunger after the procedure	44	54	51
Thirst after the procedure	38	40	31
I found it no trouble (my child did not have difficulty undergoing endoscopy)	27	19	22

EGD group (*N* = 64); CS group (*N* = 52); parents (*N* = 95).

^*∗*^N/A.
